# Supplementary material for: The interactome of intact mitochondria by cross-linking mass spectrometry provides evidence for coexisting respiratory supercomplexes
Source: Mol Cell Proteomics. 2017 Dec 8;17(2):216–32. doi: 10.1074/mcp.RA117.000470 (PMC5795388; doi:10.1074/mcp.RA117.000470)
Supplement: Supplemental Data [file supp_RA117.000470_133922_0_supp_23978_fzffwf.zip › spectra_annotation/mito_DR_spectra_annotation/21-1-1-1-12-1.pdf]

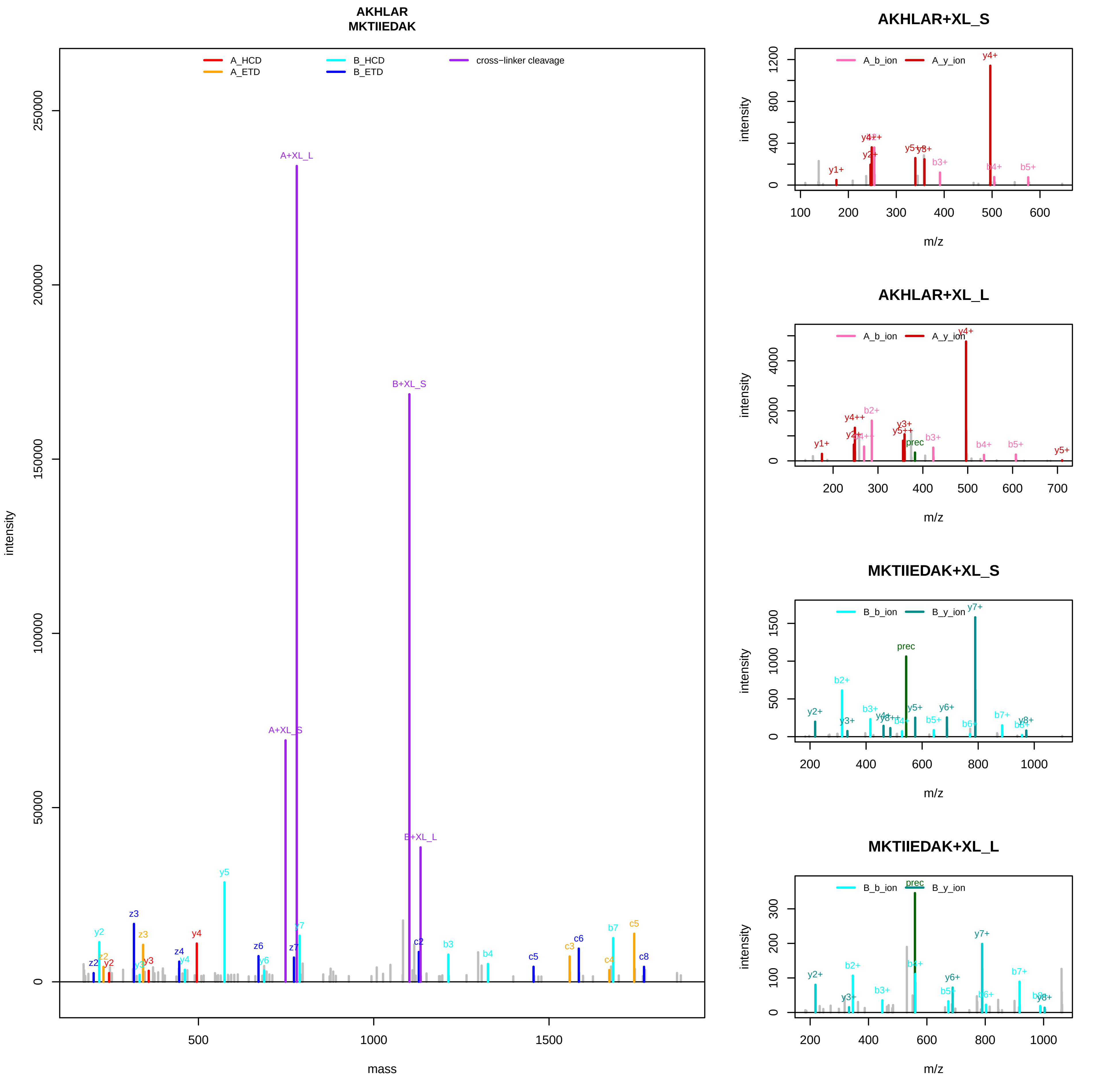
AKHLAR
MKTIIEDAK
A_HCD
A_ETD
B_HCD
B_ETD
cross−linker cleavage
A+XL_L
B+XL_S
A+XL_S
B+XL_L
intensity
mass
0
50000
100000
150000
200000
250000
500
1000
1500
y2
z2
y4
y5
z3
z4
z6
z7
y7
c2
b3
b4
c5
c3
c6
b7
c4
c8
AKHLAR+XL_S
A_b_ion
A_y_ion
y4+
y1+
b3+
b4+
b5+
m/z
AKHLAR+XL_L
y4++
b2+
prec
y5+
MKTIIEDAK+XL_S
B_b_ion
B_y_ion
y7+
y2+
y3+
b2+
b3+
y5+
y6+
b5+
b7+
y8+
MKTIIEDAK+XL_L
y6+
b7+
